# A paradigm shift in the detection of bloodborne pathogens: conventional approaches to recent detection techniques

**DOI:** 10.17179/excli2024-7392

**Published:** 2024-10-17

**Authors:** Sonali Khanal, Manjusha Pillai, Deblina Biswas, Muhammad Torequl Islam, Rachna Verma, Kamil Kuca, Dinesh Kumar, Asim Najmi, Khalid Zoghebi, Asaad Khalid, Syam Mohan

**Affiliations:** 1 School of Bioengineering and Food Technology, Shoolini University of Biotechnology and Management Sciences, Solan 173229, India; 2 Instrumentation and Control Engineering, Dr. B. R. Ambedkar National Institute of Technology Jalandhar, Punjab, 144011, India; 3 Department of Pharmacy, Bangabandhu Sheikh Mujibur Rahman Science and Technology University, Gopalganj 8100, Bangladesh; 4 Department of Pharmacy, Bangabandhu Sheikh Mujibur Rahman Science and Technology University, Gopalgonj 8100, Bangladesh; 5 Bioinformatics and Drug Innovation Laboratory, BioLuster Research Center Ltd., Gopalganj 8100, Bangladesh; 6 School of Biological and Environmental Sciences, Shoolini University of Biotechnology and Management Sciences, Solan 173229, India; 7 Faculty of Science, University of Hradec Kralove, Rokitanskeho 62, 500 03 Hradec Kralove, Czech Republic; 8 Biomedical Research Center, University Hospital Hradec Kralove, 50003 Hradec Kralove, Czech Republic; 9 Center for Advanced Innovation Technologies, VSB-Technical University of Ostrava,70800, Ostrava-Poruba, Czech Republic; 10 Department of Pharmaceutical Chemistry and Pharmacognosy, College of Pharmacy, Jazan University, Jazan 45142, Saudi Arabia; 11 Health Research Center, Jazan University, P. O. Box 114, Jazan, 82511, Saudi Arabia; 12 Center for Global Health Research, Saveetha Medical College and Hospitals, Saveetha Institute of Medical and Technical Sciences, Saveetha University, India; 13 School of Health Sciences, University of Petroleum and Energy Studies, Dehradun, Uttarakhand, India

**Keywords:** bloodborne pathogens, biosensors, rapid detection techniques, diagnosis, transmission

## Abstract

Bloodborne pathogens (BBPs) pose formidable challenges in the realm of infectious diseases, representing significant risks to both human and animal health worldwide. The review paper provides a thorough examination of bloodborne pathogens, highlighting the serious worldwide threat they pose and the effects they have on animal and human health. It addresses the potential dangers of exposure that healthcare workers confront, which have affected 3 million people annually, and investigates the many pathways by which these viruses can spread. The limitations of traditional detection techniques like PCR and ELISA have been criticized, which has led to the investigation of new detection methods driven by advances in sensor technology. The objective is to increase the amount of knowledge that is available regarding bloodborne infections as well as effective strategies for their management and detection. This review provides a thorough overview of common bloodborne infections, including their patterns of transmission, and detection techniques.

## Introduction

Microbes are ubiquitous and an integral part of our day-to-day lives. Some microbes are essential for our physiological functions. While most microbes are innocuous to us, some pathogens in nature can cause a spectrum of deadly diseases in humans (Balloux and van Dorp, 2017[11]). BBPs are harmful microbes that spread through blood and body fluids, posing a significant risk to healthcare professionals. HIV, HBV, and HCV are among the top BBPs reported globally (Mutangadura, 2004[110]). According to the World Health Organization (WHO), approximately 3 million healthcare workers are exposed to bloodborne viruses through percutaneous means. This exposure leads to an estimated annual occurrence of 16,000 cases of HCV, 66,000 cases of HBV, and 200 to 5,000 cases of HIV (Kermode et al., 2005[73]). To mitigate the risk of a pandemic and for safety and health concerns, precise detection of these pathogens is evident. Polymerase chain reaction (PCR) and enzyme-linked immunosorbent assay (ELISA) are the most used conventional techniques along with culture and colony counting methods to detect these pathogens (Lazcka et al., 2007[83]). While, these traditional methods, have been proven to be more time-consuming, complex, labor-intensive, and cost-ineffective. Biosensors, due to their ease of use, miniaturization, and real-time analysis capabilities, have gained attraction for accurate infectious illness diagnosis. Over the past three decades, biotechnological advancements have led to the development of biosensors (Vidic et al., 2017[152]). A biosensor is an assessment technique that has a molecular recognition molecule with biological origins into an appropriate physicochemical transducing mechanism and transforms it into an electrical signal from a biological response (Singh et al., 2014[139]). Bioreceptors can include various entities such as tissues, cells, enzymes, antibodies, nucleic acids, microorganisms, and organelles (Chen et al., 2020[23]). In the detection of BBPs, biosensors are commonly employed as transducing elements due to their exceptional sensitivity and accuracy. Various types of biosensors, including electrochemical, optical, microfluidics-based, and immunosensor-based biosensors, are utilized for this purpose. In addition to the traditional and advanced strategies, there are novel methods that have been effective at identifying BBPs like the electronic nose approach and aptamer approach. These non-conventional methods have also been proven to be equally specific, sensitive, flexible, and affordable over conventional techniques (Wilson and Baietto, 2011[154]; Li et al., 2020[88]).

Previous studies in the domain of BBPs reflect upon the nucleic acid diagnostics approaches and the general standardization to detect BBPs in humans and food animals. Moreover, studies ponder upon the overall pathogen detection and emphasize the traditional methods and advances in the field of diagnostics (Duncan et al., 2016[42]; Vidic et al., 2017[152]; Lazcka et al., 2007[83]; Li et al., 2020[89]). This study focuses on all the techniques used to detect BBPs with special emphasis on the recent advances in bloodborne diagnosis. This manuscript evaluates various methods for identifying bloodborne infections in humans and animals, categorizing them into conventional, non-conventional, and advanced approaches. It assesses the benefits of advanced detection methods and discusses prevalent BBPs and their transmission.

## Bloodborne Pathogens and Their Prevalence

BBPs, including viruses and bacteria, are common in blood and bodily fluids and can cause disease in humans. They can be divided into viruses, bacteria, fungi, and parasites. Examples include Dengue, West Nile, rubella, measles, EBV, HIV, HBV, poliovirus, yellow fever, and varicella-zoster virus (VZV). HIV, HVB, and hepatitis are the top three BBPs in humans (Hunter, 2017[66]; Singhal et al., 2021[140]). Bacterial BBPs are the most common source of blood-related infections. 

Some of the common bacterial BBPs are strains *Staphylococcus, Bacillus, Enterococcus*. Other than this *Listeria monocytogenes, Escherichia coli, Klebsiella pneumoniae, Haemophilus influenzae, Yersinia pestis, Francisella tularensis, and Brucella abortus*, etc. are also BBPs (Pingle et al., 2007[119]). An analysis of data from more than 200 medical facilities in 45 countries between 1997 and 2016 revealed the primary organisms that caused BSI in this survey were still E. coli and S. aureus (Diekema et al., 2019[40]). *Candida spp.*, a fungi is the second most common source of blood-linked illnesses globally (Giri and Kindo, 2012[54]). Parasites like *Cercopithifilaria, Babesia, Hepatozoon, and Theileria* are some examples of parasitic BBPs that have attacked domestic animals in the countries of the Mediterranean Basin. Also, bacterial species of *Anaplasma, Bartonella, Borrelia, Chlamydia/Chlamydophila, Coxiella, Ehrlichia, Francisella, Leptospira, Mycoplasma, Rickettsia*, and viruses from the genus Capripoxvirus, Flavivirus, and Orthonairovirus tend to attack domestic animals in this region (Defaye et al., 2022[36]). Severe animal and human infections are caused by bloodborne parasites from the genus of *Giardia, Trypanosoma, Babesia, Theileria, and Cryptosporidium*. Additionally, the morbidity and mortality of animals have been linked to parasites of the genus *Hepatozoon* (Barbosa et al., 2017[13]). According to a study in Egypt, several pathogens affect food animals, including strains of *Theileria, Borrelia, Anaplasma, Babesia, Coxiella* (Abdullah et al., 2021[1]). The various BBPs prevailing in humans and food animals are enlisted in the Supplementary information (Table S1 and S2), respectively.

### Transmission of bloodborne pathogens to humans and food animals

**Figure 1:**
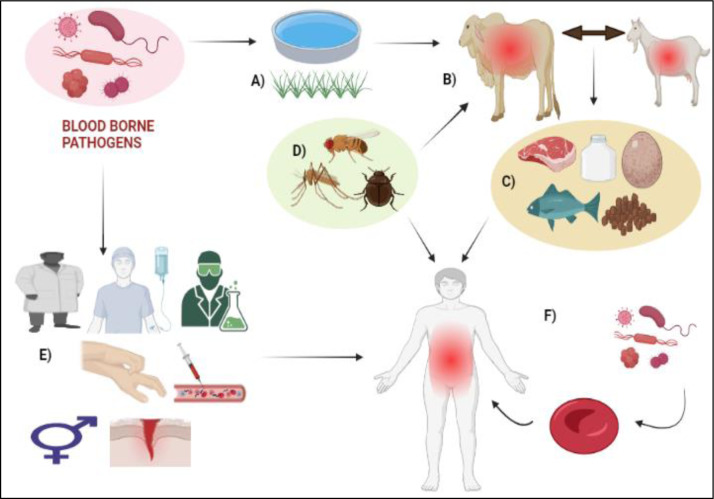
Transmission cycle of bloodborne pathogens (A) contaminated water and food B) consumption of water and food by animals, C) consuming food products like milk, meat, eggs, etc. produced from infected animals, D) insect vectors transmit bloodborne pathogens in humans as well as food animals, E) direct contact between healthcare professionals and infected patients, F) penetration of bloodborne pathogen in the bloodstream causes infectious diseases like AIDS, Hepatitis, Dengue, etc.

BBPs are microorganisms that can cause severe diseases when transmitted through the bloodstream. Bloodborne infections (BBIs) are the term used to describe the infection caused by the BBPs. Healthcare professionals are at a significant risk of exposure to BBPs, which can be spread through mucocutaneous contact, percutaneous damage, unintentional punctures, bites, cuts, and abrasions (Figure 1(Fig. 1)). Factors such as viral load, injury exposure, and recipient immune status influence the spread of BBPs. In Zambian healthcare, the average rate of sharp injuries per respondent is eight times higher than in the United States, indicating a higher risk of exposure to BBPs (Denault and Gardner, 2022[37]; Beltrami et al., 2000[14]; Lanphear, 1994[81]; Pirozzolo and LeMay, 2007[121]; Xeroulis et al., 2005[156]; Deuffic-Burban et al., 2011[38]; Cleveland et al., 2016[28]; Phillips et al., 2012[118]).

Food-producing animals (such as cattle, chickens, pigs, and turkeys) are the main reservoirs for several pathogens (EFSA, 2016[44]). Food-producing animals play an important role in pathogen transmission; for example, beef is claimed to have been the cause of 7 % of the 1.7 million cases of foodborne illness documented in England and Wales between 1996 and 2000 (Anderson et al., 2009[4]). Food animals, raw or undercooked meat, contaminated water, and food-processing equipment are potential sources of transmission for the Hepatitis E virus (HEV), the leading cause of enteric viral hepatitis infection globally. In Europe, domestic swine herds often exhibit high prevalence rates of HEV, making it a significant global health concern (EFSA, 2017[45]). Animals can also get ailments besides functioning as BBP transmission vectors. Transmission of BBPs in humans and animals is passed on via a varied scale of pathogens spread by arthropod routes (Figure 1(Fig. 1)) (Abebe et al., 2020[2]; Baneth, 2014[12]). Food animals such as sheep, goats, pigs, and chickens can potentially acquire *Toxoplasma gondii* infections. Tissue cysts are present in diseased animals, and consumption of these cysts in undercooked or raw meat can cause infection in humans. *Tactyzoites *found in blood products, tissue transplants, and unpasteurized milk may also be a source of transmission (Hill and Dubey, 2018[61]). Numerous species of hard ticks infest cattle, and these ticks can transmit several pathogenic diseases like bovine babesiosis, which is brought on by *Babesia* *bigemina *and *Babesia bovis*; bovine anaplasmosis, which is brought on by *Anaplasma marginale*; and heartwater, which is brought on by *Ehrlichia ruminantium* (Aouadi et al., 2017[6]).

The BBP located in the blood and skin of the animals that serve as its hosts are mechanically transported by the *Stomoxys* fly. It may spread bacteria like *Trypanosoma*, *Besnoitia*, and *Rickettsia* as well as viruses including African swine fever, Rift Valley, West Nile, and equine infectious anemia (Hailemariam et al., 2017[59]). Chagas disease (CD) is brought on by the protozoan parasite *Trypanosoma* *cruzi* (*T*. *cruzi*). It can be blowout by blood transfusions, organ transplantation, incidental blood exposure, eating food contaminated with infected triatomine insects, or any other situation where blood-sucking triatomine insects are present (Bern et al., 2011[15]; Jankowska et al., 2020[70]).

### Detection methods of bloodborne pathogens

BBPs screening is crucial for preventing cross-infections and diseases transferred through blood transfusions. Biosensors are appealing analytical tools for rapid and accurate infectious disease detection because of their simplicity, ability to be miniaturized, and capacity for real-time analysis. Over the past 30 years, several biotechnological developments have created biosensors intended for the recognition and checking of pathogens (Vidic et al., 2017[152]). Advanced biosensing technologies, including aptamer-based and electronic nose techniques, have proven effective in identifying BBPs, offering quick screening for infectious illnesses and high stability (Park, 2018[115]; Turner and Magan, 2004[149]). The diagnostic methods used currently in clinical settings for detecting BBP in humans are mentioned in Table 1(Tab. 1) (References in Table 1: Camejo Leonor and Mendez, 2024[18]; Dunn et al., 2020[43]; Fischer et al., 2017[50]; Kabir et al., 2021[71]; Kham-Kjing et al., 2022[74]; Pan et al., 2023[113]; Patel and Cassidy, 2018[116]; Puri et al., 2022[123]; Silva et al., 2019[136]; WHO, 2017[153]; Xie et al., 2021[158]; Xu et al., 2024[161]).

## Conventional Approaches for the Detection of Bloodborne Pathogens

### PCR-based approaches to detect bloodborne pathogens

PCR is a laboratory enzymatic process that enables the amplification of short DNA segments. In PCR, target DNA is replicated using a thermostable DNA polymerase enzyme in the presence of nucleotides and primers (Yang and Rothman, 2004[163]). PCR plays a crucial role in molecular diagnostics as it allows for the sensitive and specific amplification of nucleic acids, making it a significant technique in molecular diagnostics and various fields of biological sciences (Miao et al., 2020[105]). Schijman et al. (2011[129]) conducted a groundbreaking investigation using blood samples from 32 seropositive and 10 seronegative patients to investigate blood infections brought on by the *T. cruzi* virus in patients from South Cone countries. They identified *T. cruzi* DNA at concentrations as low as 10 fg/microliter and 0.5 parasites/ml using Polymerase Chain Reaction (PCR) techniques, with sensitivity ranging from 83.3 % to 94.4 % and specificity ranging from 85 % to 95 %. With the potential to improve diagnostic capacities and disease management tactics in the fight against Chagas disease, this ground-breaking research marked a significant turning point in the global validation of PCR techniques for reliably detecting *T. cruzi* in human blood samples. Contini et al. (2005[29]) developed and evaluated a highly sensitive real-time PCR (Light-cycler, LC-PCR) to detect and quantify *T. gondii* B1 and bradyzoite-specific genes (SAG-4, MAG-1) in serum and PBMC specimens from immunocompetent subjects with or without suspected *T. gondii *infection. The LC-PCR targeting the B1 gene showed high sensitivity, detecting quantities as low as 10^2^ to 10^-3^ parasites/ml. Cox et al. (2005[31]) collected field samples, including 245 samples of bovine blood drawn from communities in Uganda's Soroti and Tororo regions. Positive amplification was achieved even with DNA concentrations as low as 55 pg/ml, indicating the sensitivity of the PCR assay in detecting trypanosomes. However, the clinical application of PCR assays has certain limitations, including the potential for false-negative and false-positive results, background DNA contamination leading to false positives, detection sensitivity exceeding clinical significance, and limited detection capacity for simultaneous identification of multiple species, virulence factors, or drug resistance (Chen et al., 2013[21]; Schijman et al., 2011[129]; Contini et al., 2005[29]; Cox et al., 2005[31]). Table 2(Tab. 2) (References in Table 2: El-Dakhly et al., 2020[47]; Grabias et al., 2019[55]; Grigorenko et al., 2017[57]; Hsia et al., 2007[62]; Lawal et al., 2021[82]; Mansuy et al., 2018[102]; Mohmad et al., 2018[108]; Pripuzova et al., 2012[122]; Saadat et al., 2017[126]; Sharma et al., 2022[131]; Spiess et al., 2007[144]; Tsegay et al., 2022[148]; Udonsom et al., 2022[150]; Zhang et al., 2018[170]) describes the application of PCR to BBP detection.

### ELISA-based approaches to detect bloodborne pathogens

ELISA is a gold standard immunoassay used to detect and measure molecules like antibodies, antigens, proteins, glycoproteins, and hormones. It can identify biological processes quickly and cost-effectively, and detect significant infections. Antibodies are complexed with antigens, yielding quantifiable outcomes. The analyte can be any protein or a complex combination of proteins (Konstantinou, 2017[79]; de la Rica and Stevens, 2012[35]). 

The ELISA test has been utilized in peptide and protein analysis a lot lately. This test is sensitive and focused, and it yields findings quickly. Due to its simplicity and quickness, it may be used in a wide range of situations. Additionally, it is more efficient because only one serum sample needs to be examined. The ELISA test does not need specialized tools or radioactive labeling, although it is virtually as sensitive as radio-immunoassays (RIA). However, compared to RIA, its dependability is poor (Aydin, 2015[8]). To investigate the biological importance of NS1 secretion *in vivo* and detect the presence of this protein in the sera of DENV-infected patients, Alcon et al. (2002[3]) developed a highly sensitive ELISA assay. The sensitivity of the ELISA was demonstrated to be less than 1 ng/ml when using pure dengue virus type 1 NS1 as a protein standard. Noedl et al. (2006[112]) collected a total of 700 whole blood samples from symptomatic outpatients of malaria clinics located along the Thai-Myanmar border. The HRP2 ELISA showed an overall sensitivity of 98.8 % (95 % CI, 93.6-100 %) and a specificity of 100 % (95 % CI, 99.5-100 %) for detecting *P. falciparum malaria* (Alcon et al., 2002[3]; Noedl et al., 2006[112]). Table 2(Tab. 2) describes the application of ELISA to BBP detection.

### Array-based approaches to detectbloodborne pathogens

DNA microarrays are inverse dot blots in which a substrate is coupled to a lattice of ''probes'' with predetermined sequences. The final picture displays probes as "spots," where each spot represents a distinct probe sequence. Typically, spots are 200 to 500 micrometers (µm) apart and 100 to 200 micrometers (µm) in size. The array is loaded with targets, and any reporter molecules are employed to detect targets that combine with complementary probes. These later ones are either mechanically attached to the substrate or lithographically constructed on the spot. PCR by-products or oligonucleotides are used as probes (Call et al., 2003[17]). Microbial diagnostic microarrays (MDMs) utilize three types of probes: (I) short oligonucleotides, (II) long oligonucleotides, and (III) PCR amplicons. Short oligonucleotides often contain 1-2 mismatches, which can limit their binding ability and require the use of PCR amplification to enhance specificity. Stronger binding capacities can be used with more universal amplification techniques (like WGA-whole genome amplification), even though the discriminating ability of long oligonucleotide probes and PCR products is low (80-85 % sequence homology). Hybridization specificity is significantly influenced by the position of the mismatch in addition to probe length (Letowski et al., 2004[86]). The conventional microarray format typically consists of a flat glass slide that undergoes various surface modifications to facilitate the covalent attachment of probe molecules in research laboratories. However, this approach has drawbacks such as cost inefficiency and complex implementation across the organization. Table 2(Tab. 2) describes the application of array-based methods to BBP detection. 

## Advanced Approaches to Detect Bloodborne Pathogens

### Biosensor-based approaches to detect bloodborne pathogens

**Figure 2:**
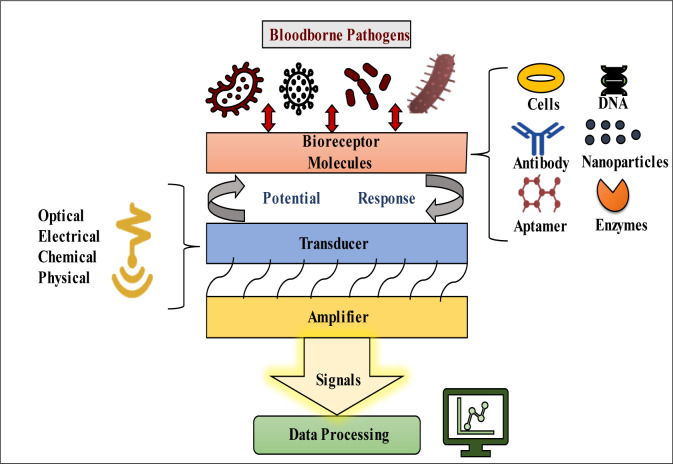
A schematic diagram illustrating the working principle of a biosensor

A biosensor is an analytic tool that uses a transducer to transform biological recognition of a target analyte into a quantifiable signal (Figure 2(Fig. 2)). The glucose sensor for the control of diabetes is the most well-known application of these sensors in the modern environment (Sin et al., 2014[137]). Lateral flow assays, such as home pregnancy tests, are also being extensively employed. The biosensor is not only an easy-to-use technique, but it is also an inexpensive technology with high sensitivity and specificity to rapidly identify pathogens that cause infectious diseases (Foudeh et al., 2012[51]). Biosensors are required to easily detect pathogens that cause emerging infectious diseases (EIDs). The analytical sensitivity of the device can easily detect very low levels of antigens without significant changes in selectivity. A biosensor is a cheap and robust technique that is highly desired for field applications since it provides high throughput (Pejcic et al., 2006[117]). In their study, Gray et al. (2018[56]) utilized an inexpensive component found in cell phones to identify HIV in 133 individuals. The biosensor exhibited remarkable sensitivity, specificity, low sample volumes, and rapid results. Testing different biomarkers in 102 healthy volunteers and 31 plasma samples from HIV patients, the biosensor achieved a combined sensitivity of 100 % for anti-gp41 and 64.5 % for anti-p24, with 100 % specificity, all within a 5-minute timeframe. Another study focused on a multiplex biosensor capable of identifying pathogens in physiological samples by detecting species-specific bacterial 16S ribosomal RNA sequences without the need for preamplification. The viability of this biosensor for pathogen identification and rapid diagnosis of bloodstream infections was investigated. The electrochemical biosensor demonstrated complete agreement with microbial analysis, successfully detecting various types of microbes present in the tested samples (Gray et al., 2018[56]). Different types of biosensors are typically briefed in Table 3(Tab. 3) (References in Table 3: Andersson et al., 2020[5]; Atias et al., 2009[7]; Babamiri et al., 2018[9]; Chaudhary et al., 2021[20]; Chen et al., 2019[24]; Chuang et al., 2020[27]; Darwish et al., 2016[34]; Faria and Mazon, 2019[48]; Hemben et al., 2017[60]; Huang et al., 2016[65]; Kong et al., 2019[78]; Liu and Huang, 2012[94]; Lo et al., 2021[95]; Luz et al., 2015[98]; Martins et al., 2020[103]; Medyantseva et al., 2000[104]; Ming et al., 2015[106]; Mohammed et al., 2021[107]; Rahi et al., 2016[124]; Sharma et al., 2010[130]; Song et al., 2016[142]; Xiang et al., 2013[157]; Zhang et al., 2010[169]; Zhao et al., 2019[171]; Zheng et al., 2012[172]; Zhi et al., 2014[173]; Zhou et al., 2014[174]) and Table 4(Tab. 4) (References in Table 4: Biagetti et al., 2018[16]; Cordeiro et al., 2019[30]; Li et al., 2019[90]; Manessis et al., 2022[101]; Saleh and El-Matbouli, 2015[127]; Santos et al., 2012[128]; Silva et al., 2006[134], 2008[135]; Vázquez-Guardado et al., 2021[151]; Wu et al., 2020[155]; Yang et al., 2014[162], 2018[164]; Ye et al., 2019[165]) with the technology specification.

#### Electrochemical biosensor to detect bloodborne pathogens

The fundamental principle of an electrochemical biosensor is to convert biological events, such as enzyme-substrate interactions and antigen-antibody interactions, into electrical signals, such as current, voltage, or impedance (Cho et al., 2020[26]). To obtain valuable information regarding flaviviral infections (such as DENV, ZIKV, and JEV), various electrochemical detection methods have been developed and utilized. These methods include potentiometry (measuring the potential of an indicator electrode), conductometry (measuring conductivity or resistance) and amperometry/voltammetry (measuring current as a function of imposed potential) (Khristunova et al., 2020[76]). The combination of amino-reduced graphene oxide (NH_2_-rGO) and β-cyclodextrin (-CD) was utilized to modify the surface of a glassy carbon electrode (GCE) to detect the HIV gene using differential pulse voltammetry (DPV). The electrochemical biosensor exhibited excellent sensitivity and selectivity, achieving a limit of detection (LOD) of 8.7 fM. The electrochemical biosensor was developed by Li (2020[89]) for detecting the HIV segment in human serum samples, demonstrating successful detection of the target using electrochemical methods (Li, 2020[89]). To detect the Listeria hlyA gene, an impedimetric biosensor was created. It worked by immobilizing a DNA-detecting probe on a poly-5-carboxy indole (5C Pin) polymer. When it came to target DNA concentration, this label-free biosensor showed a detectable linear range of 1 × 10^-4^ to 1 × 10^-12 ^M (moles per liter). After being covalently immobilized on the 5C Pin polymer, the target DNA sequence underwent further hybridization with the probe. Charge transfer resistance, expressed in ohms (Ω), was used to assess the biosensor's performance and provide information on changes in electrical impedance at the electrode interface. With potential uses in clinical diagnostics and food safety, this novel method shows promise for the extremely sensitive and specific identification of Listeria hlyA gene sequences (Kashish et al., 2015[72]). Electrochemical biosensors provide quick, precise, and sensitive results at an affordable cost. Nanomaterials and nanocomposites enhance sensitivity. Microfluidic systems integrate with biosensors for miniaturized platforms. Challenges include stability, repeatability, low LOD values, and sample sensitivity (Singh et al., 2021[138]). 

#### Optical biosensor to detect bloodborne pathogens

Optical biosensors, including colorimetric, fluorescence, SPR, optic fibers, and biological luminescence, are compact diagnostic devices used for pathogen detection, generating electrical signals (Damborský et al., 2016[32]; Eksin and Erdem, 2023[46]). These provide an alternative technique for viral detection, offering reliability, user-friendliness, and cost-effectiveness. They also reduce the reliance on nucleic acid amplification. These biosensors have been used to identify pathogens such as HIV, Ebola, norovirus, and influenza virus, utilizing techniques like fluorescence, surface plasmons, and colorimetry. Nano-biosensors enable targeted and single-virus scanning for infection detection (Maddali et al., 2020[99]). Colorimetric biosensors utilize ligand-target interactions that result in a visible color change, enabling simple and portable optical quantification (Piriya VS et al., 2017[120]). To quantify fluorescence, a fluorophore substance must be activated at one wavelength to produce light emission at another wavelength. Fluorescent dye-labeled reporter molecules provide for sensitive analyte detection (Li et al., 2013[87]). A fluorescent biosensor for HBV DNA sequence recognition based on gold nanorods (AuNRs) was created by Lu et al. (2013[96]). By mixing fluorescein-tagged single-stranded DNA (FAM-ssDNA) with the AuNRs solution, they produced a ternary complex known as FAM-ssDNA-CTABAuNRs. According to Lu et al. (2013[96]), this led to fluorescence resonance energy transfer (FRET) from FAM to AuNRs, which decreased the fluorescence intensity of FAM. One effective method for identifying molecular species at the single-molecule level is surface-enhanced Raman scattering (SERS). The sensitivity and chemical specificity of current optical detection techniques can be substantially exceeded by attaching molecules to nanostructured substrates, hence increasing the Raman signal (Lin et al., 2023[91]). An essential aspect of surface-enhanced Raman scattering (SERS) is surface plasmon resonance (SPR), which takes place at the interface between a metal and a dielectric. SPR occurs when longitudinal electron waves, induced by light that is polarized parallel to the surface, cause a concentration of oscillating electrons (Lazcka et al., 2007[83]). Liu and Huang (2012[94]) used DNA-silver nanoparticle (AgNP) conjugates in a range of 0.30 to 2.0 nmol/L to quantitatively detect HIV DNA using a sandwich approach based on SPR (Liu and Huang, 2012[94]). To detect changes in mass on the probe/transducer surface, piezoelectric sensors that employ quartz crystal microbalances (QCM) measure resonance frequency fluctuations (Lazcka et al., 2007[83]). 

#### Aptamer-based biosensor to detect bloodborne pathogens

Aptamer-based biosensors, also known as aptasensors, are biosensors that use aptamers for identification. They are versatile, can be easily changed, and have predictable secondary structures. They are effective in detecting blood infections and are known for their rapid detection sensitivity due to their integration with signal amplification techniques (Liu et al., 2021[93]). However, the absence of excellent aptamers for therapeutically significant targets is one of the field's current constraints. To enhance its accuracy and dependability, the aptamer technology must also undergo comprehensive testing in a clinical sample matrix (Zhou et al., 2014[175]). A simple method was devised to construct aptamer-based fluorescence biosensors for the quantitative measurement of Hepatitis B antigen (HBeAg). The molecular detection element in this technique is an HBeAg aptamer that has been fluorescence-labeled, and a tiny DNA molecule that is similar to the aptamer is used as a competitor. It is reported that this approach yields a limit of detection (LOD) for HBeAg of 609 nanograms per milliliter (ng/ml). Fluorescence tests were performed on blood serums that were positive and negative for HBeAg after this biosensor was established, and statistical significance was noted (p < 0.05). Notably, it just takes two minutes to finish the detection test in its whole. These recently identified aptamers have the potential to enhance the chronic hepatitis B diagnosis process (Huang et al., 2016[65]). Furthermore, a different aptamer-based biosensor was created with good specificity for the diagnosis of the Hepatitis C virus (HCV) and the identification of the virus core antigen in infected individuals. A random library of 60 RNAs, consisting of roughly 10^15^ sequences, was used to generate high-affinity aptamers using the systematic evolution of ligands by the exponential enrichment (SELEX) approach. Subsequent protein chip tests revealed that the chosen aptamers bound to the HCV core antigen selectively, but not to the other HCV antigen, NS5. The concentrations and the limit of detection are accurately represented in nanograms per milliliter (ng/ml), giving a clear picture of the sensitivity of the detection. These biosensors based on aptamers have a great deal of promise to improve HCV infection detection and treatment (Lee et al., 2007[85]).

#### Immunosensor to detect bloodborne pathogens

Affinity solid-state-based biosensors, known as immunosensors, are utilized to monitor specific analytes, such as antigens (Ag), by establishing a stable interaction between the Ag and an antibody (Ab) that acts as the binding agent. This immune reaction generates a measurable signal through a transducer, as described by Gil Rosa et al. (2022[53]). Using highly specific antibody-antigen bond, a variety of biomolecules, including viruses, bacteria, protein markers, nucleic acids, and other tiny particles, are identified (Mahato et al., 2018[100]). According to one of the investigations, a gold film electrode from a recordable compact disc (CD-trade) was used to create an electrochemical immunosensor for the NS1 protein (Cavalcanti et al., 2012[19]). The immunosensor responded linearly to NS1 concentrations between 1 and 100 ng/ml, with a 0.33 ng/ml detection limit. With a detection limit of 200 fg/ml, a highly sensitive label-free immunosensor was created for the detection of HIV-1. It is a potential strategy for viral identification and evaluation of biological and medicinal samples (Lee et al., 2013[84]). Additionally, a completely novel amperometric immunosensor for the HIV-1 p24 antigen (p24Ag) detection was found. With a relatively low detection limit of 0.0064 ng/ml (S/N = 3), the predicted immunosensor demonstrated good electrochemical sensitivity to the presence of p24 in a concentration range of 0.01 to 60.00 ng/ml (Kheiri et al., 2011[75]). An ultrasensitive immunosensor for measuring hepatitis B surface antigen at a detection limit of 0.36 pg/ml exists, according to one of the assessments. Hepatitis B surface antigen was detectable by the immunosensor in the linear concentration range of 1.7 to 1920 pg/ml (Shourian et al., 2015[133]). Immunosensors have a significant deal of potential to develop into effective measuring devices because of their real-time estimation, constrained sample intake, relatively inexpensive price, and easy apparatus operation (Janik-Karpinska et al., 2022[69]). 

#### Microfluidics-based biosensor to detect bloodborne pathogens

Microfluidic biosensors are integrated chip devices that combine various activities, offering reduced reagent and specimen consumption, flexible liquid manipulation, and reduced detection times due to their integration with characterization techniques (Xing et al., 2022[160]). Many microfluidic LOC devices have been used in a variety of assays and biosensors, such as paper microfluidic assays, magnetic bead cell sorting assays, particle immunoassays, and microfluidic ELISAs for pathogen detection. These advancements have raised the levels of specificity, sensitivity, usability, and mobility (Fronczek, 2013[52]). There are many instances where microfluidic approaches have proven to detect pathogens well. According to Huang and Huang (2019[64]), SERS may be utilized to quickly identify *E. coli* strains that cause sepsis when combined with microfluidic microwell technology. Conventional detection methods don't require long culture durations since the bacteria in the microwell may be contained there, increasing the effective bacterium concentration by up to 10^7 ^folds (Huang and Huang, 2019[64]). Micro- and nanotechnologies have permitted clear advances in HIV diagnosis, with the commercial availability of microfluidics-based CD4+ T cell counts utilizing a drop of blood from a finger prick (Damhorst et al., 2015[33]). In contrast to conventional methods, the employment of fluorescent microsphere-based lateral flow immunoassay strips (FM-LFIAs) for standard swine fever (CSFV) detection yielded a sensitivity of 5.28 ng/ml, covering a linear range of values from 9.77 to 625 ng/ml (Xie et al., 2020[159]). Ikeda et al. (2009[67]) showed how to use an on-chip flow cytometer and a disposable microfluidic device to detect Listeria monocytogenes (pathogenic bacteria) in milk (Ikeda et al., 2009[67]). Hsieh et al. devised a nanofluidic pre-concentrator and a nanoslit Fano resonance biosensor to identify latent membrane protein 1 (LMP1) for diagnosing Epstein-Barr virus (EBV). The cost-effective nano-slit plasmonic sensor chip can be produced on a large scale using nanoimprinting and aluminium deposition. The nonporous membrane was designed as an ion-selective channel integrated into the sensor chip to enhance the concentration of LMP1 proteins. Subsequently, a sensor chip employing anti-LMP1 IgG was developed to detect LMP1, achieving a limit of detection (LOD) of 100 pg/ml, while the sensing range spanned from 100 pg/ml to 10 g/ml. (Hsieh et al., 2022[63]). Studies show microfluidics offer accurate, convenient, and quick live pathogen detection techniques. However, they lack sensitivity and have limited capacity for high sample quantities. Obstacles like usability, cold storage, and electricity-free operations need to be addressed for low-resource applications (Spatola Rossi et al., 2023[143]). 

### Non-biosensing technology to detect bloodborne pathogens

#### Aptamer-based approaches to detect bloodborne pathogens

Aptamers are molecules having properties in a fashion like antibodies with complex 3-D structures. These single-strand nucleic acids (DNA, RNA) work by forming an affinity bond with their target molecules. Due to their increased stability, aptamers, which are typically 22-100 nucleobases in length, are capable of denaturation and renaturation. The variable region edged by constant regions in the aptamers allows the amplification and identification of sequences (Devi et al., 2021[39]; Sharma and Shukla, 2014[132]). Aptamers have been employed for pathogen detection and concentration applications because of their excellent specificity and affinity in binding to their target, which is dependent on the main sequence which is described in Table 5(Tab. 5) (References in Table 5: Bachour Junior et al., 2021[10]; Figueroa-Miranda et al., 2020[49]; Mohsin et al., 2020[109]; Rahmati et al., 2021[125]; Tang et al., 2016[146]; Zhu et al., 2012[176]) (Nagarkatti et al., 2014[111]). The detection and recognition of targets rely on factors such as the length of nucleic acid molecules within aptamers, the primary sequence, and various environmental variables. In addition, the hydrogen bonds, the forces of van der Waals, hydrophobic contacts, and the combination of complementarity in form all contribute to the intermolecular interaction that occurs between the target and aptamers. As a result, the aptamers gain distinctive qualities such as *in vitro* creation, stable temperature, pH intervals, non-toxicity, and better immune response (Yue et al., 2021[167]).

#### Electronic nose-based approaches to detect bloodborne pathogens

An electronic nose (E-nose) is a device with chemical sensors that mimic human olfactory perceptions. It identifies volatile organic molecules with varying specificity and uses a signal-pre-processing unit and pattern recognition system. The gas sensor array converts molecular signals into electrical signals, generating unique odor patterns. E-nose offers rapid sensing and less bias, providing more consistent measurements between devices (Chen et al., 2013[22]; Ye et al., 2021[166]; Kumar et al., 2020[80]; Tan and Xu, 2020[145]). An illustration of the concept of utilizing an electronic nose to identify multiple categories of volatile organic compounds (VOCs) is present in the gas phase of blood samples. In this investigation, sera from 24 control cattle from para-TB non-suspect herds and sera from 43 dairy cattle with spontaneously occurring para-TB infections were obtained. Additionally, 24 sera from control cattle that were not infected and 26 sera from naturally brucellosis-infected animals were collected. At the population level, the e-nose could only discriminate sera from brucellosis and paratuberculosis-infected animals from healthy animals. The study's findings therefore demonstrate the potential of VOC analysis for the distinction of viral illnesses in animals, despite the limitations of the technology. Also, disease-specific volatiles must be found utilizing techniques like gas-chromatography. It is necessary to develop an e-nose approach so that VOC analysis can eventually be used as a cutting-edge diagnostic test (Pardo and Sberveglieri, 2010[114]).

## Detection Challenges of Bloodborne Pathogens in Humans and Food Animals

Due to the characteristics of these bloodborne pathogens (BBPs) and the difficulty of detection techniques, detecting infections caused by them in humans and animals involves several difficulties (Dong et al., 2008[41]). Acquiring suitable samples for testing can be difficult, particularly when it comes to BBI and it may be necessary to perform intrusive operations that put the patient in danger or discomfort, such as blood draws or tissue biopsies (Smalls and Fischbach, 1982[141]). During the window period, the viral load is low, and antibodies or antigens may not be detected, resulting in false-negative findings (Taylor et al., 2014[147]). The accuracy of diagnostic testing is impacted by viral mutations in conditions like human immunodeficiency virus (HIV) infections (Guatelli et al., 1989[58]). Cross-reactivity occurs when antibodies or antigens that are identical to the target pathogen are present in diagnostic testing, leading to false-positive findings (Klarkowski et al., 2013[77]). Several BBPs, like HIV, HCV, and HBV, can develop chronic infections without exhibiting symptoms for a protracted length of time. As a result, afflicted people can go untreated and unwittingly spread the infections to others (Ludlam et al., 2006[97]). Food animals can contract bloodborne pathogens like Salmonella or the bovine viral diarrhea virus, causing persistent infections without visible symptoms. Multiple diseases can co-infect food animals, making diagnosis more challenging. Sensitivity and specificity are crucial for accurate detection of BBPs in humans and animals. However, accessibility and availability of diagnostic tests can be constrained in resource-constrained areas or during public health emergencies (Jain et al., 2021[68]; Chen et al., 2014[25]; Zadran et al., 2022[168]).

## Conclusion and Recommendations

BBPs pose serious threats to the welfare of food animals as well as to human health due to their adverse effects. Through a variety of mechanisms of transmission, these viruses can enter circulation and cause various life-threatening infectious illnesses, known as BBIs. To maintain safety and stop the spread of illness, early detection of BBIs is essential. For the detection of these BBPs, techniques like PCR, ELISA, and array-based analysis have been used historically; nevertheless, they come with complexity and time-consuming requirements. However, the non-conventional methods have become very specific and sensitive substitutes for the identification of BBPs. Among these, biosensor technology is a particularly innovative means of detecting BBP. Biosensors based on optical, microfluidic, and immunosensor technologies have proven to be more sensitive and precise than others, and they also take less time and expenditure to operate. Future developments in biosensing technologies could potentially lead to improved BBP detection performance. The development of biosensors that are optimized for incredibly accurate BBP detection needs to be the top priority for future research projects. To successfully advise researchers, this entails addressing major knowledge gaps in the creation of biosensors and taking important references in BBP detection into consideration. Furthermore, the development of the next generation of biosensing technology, which may result from the integration of diverse biosensing approaches would allow for immediate and thorough monitoring of BBPs. The necessity of transferring lab-scale research into large-scale industrial output must be made widely known. To achieve the highest standards of safety for consumers and healthcare professionals, as well as to ensure the safety of food animals, this transformation is essential. Overall, the continued advancement and adoption of biosensor technology offer promising prospects for early and efficient detection of BBPs. By harnessing the capabilities of biosensors and addressing key challenges, we can significantly enhance our ability to monitor and manage infectious diseases, ultimately safeguarding public health worldwide.

## Notes

Rachna Verma and Asaad Khalid (Health Research Center, Jazan University, P. O. Box 114, Jazan, 82511, Saudi Arabia; E-mail: drasaad@gmail.com) contributed equally as corresponding author.

## Declaration

### Data availability

All data related to this study has been included in the manuscript.

### Declaration of interest

The authors declare that they have no known competing financial interests or personal relationships that could have appeared to influence the work reported in this paper.

### Funding

The authors gratefully acknowledge the support of the Deanship of Graduate Studies and Scientific Research, Jazan University, Saudi Arabia, for this research through the Research Funding Program (Project Number: RG24-L05).

### Ethical approval

Not applicable. 

### Consent to participate

Not applicable. 

### Research involving human participants and/or animals

Not applicable.

## Supplementary Material

Supplementary information

## Figures and Tables

**Table 1:**
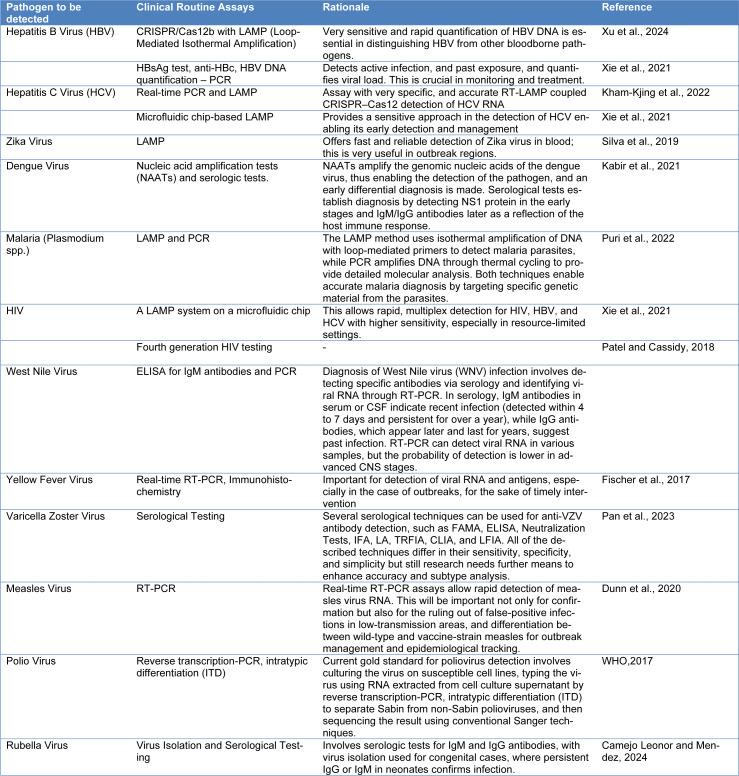
Current clinically used diagnostic techniques for the detection of bloodborne pathogens in humans

**Table 2:**
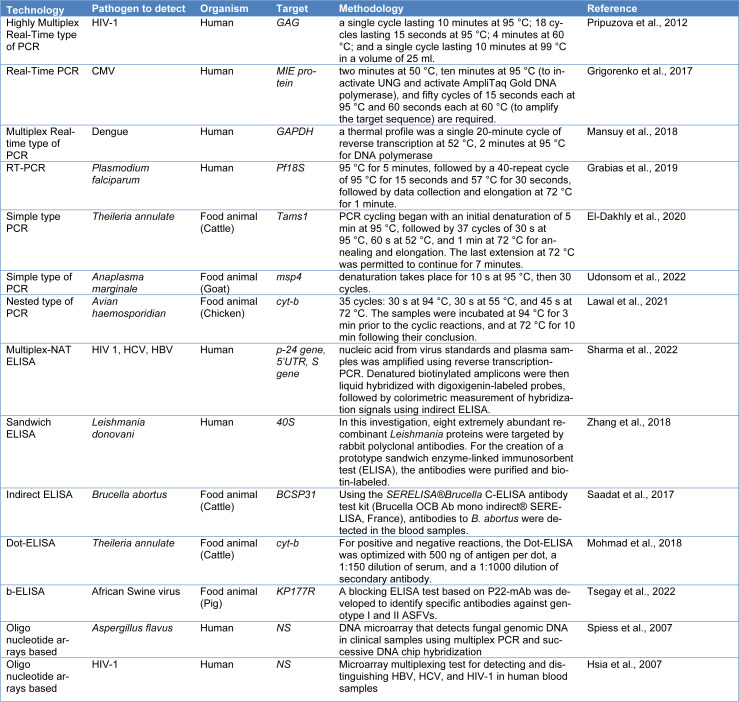
Conventional approaches to detect bloodborne pathogens in humans and food animals

**Table 3:**
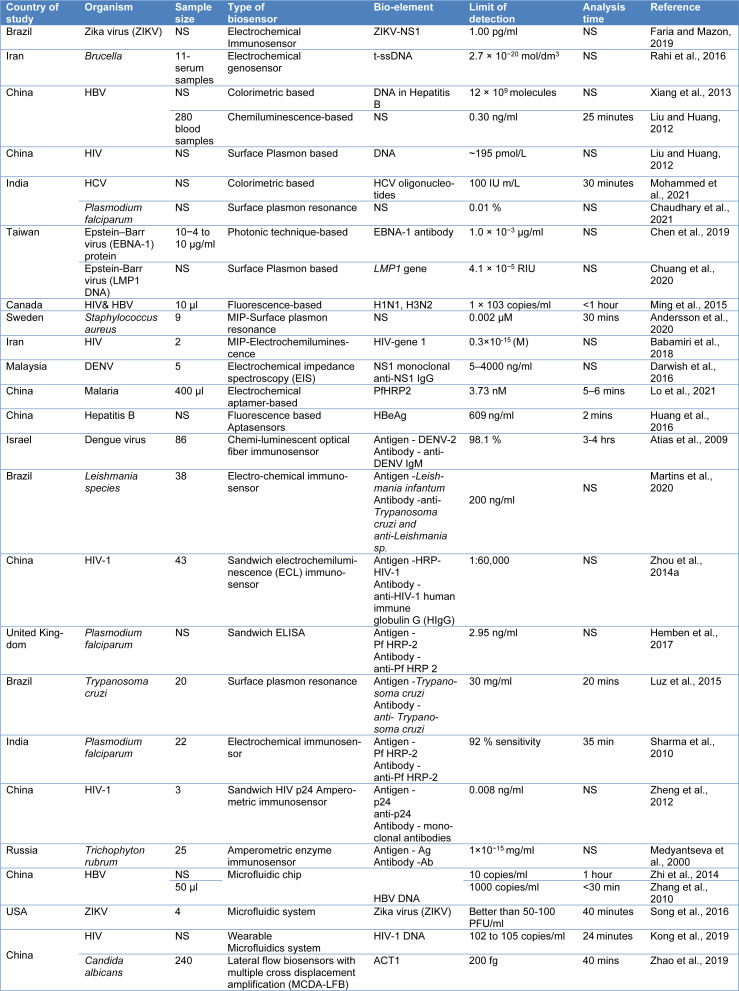
Currently used biosensors to detect bloodborne pathogens in humans

**Table 4:**
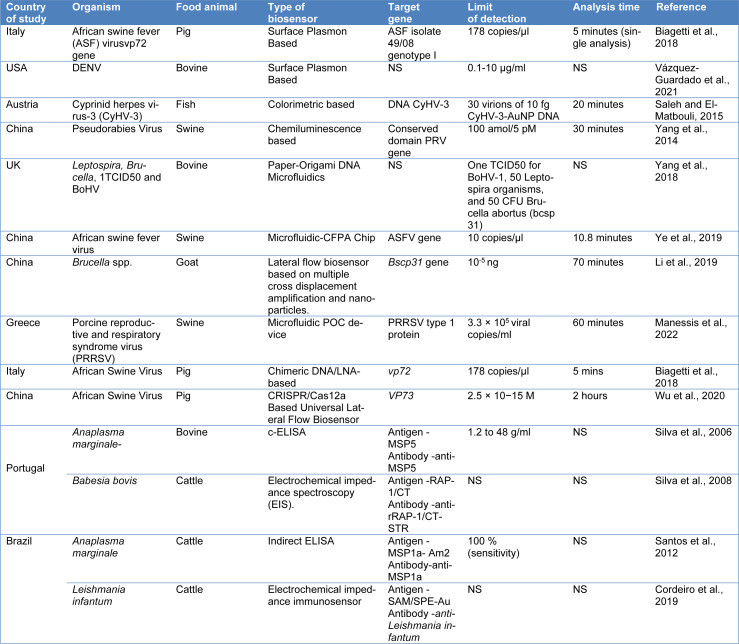
Currently used biosensor to detect bloodborne pathogens in food animals

**Table 5:**
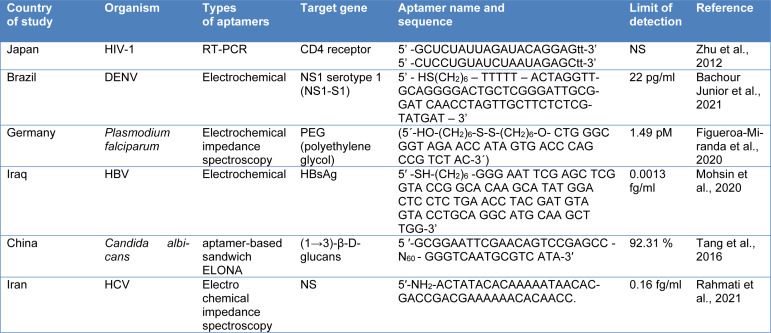
Aptamer-based techniques to detect bloodborne pathogens
