# Supplementary material for: A paradigm shift in the detection of bloodborne pathogens: conventional approaches to recent detection techniques
Source: EXCLI J. 2024 Oct 17;23:1245–75. doi: 10.17179/excli2024-7392 (PMC11579516; doi:10.17179/excli2024-7392)
Supplement: Supplementary information [file EXCLI-23-1245-s-001.pdf]

EXCLI Journal 2024;23:1245-1275 – ISSN 1611-2156
Received: May 15, 2024, accepted: September 04, 2024, published: October 17, 2024

Supplementary information to:

Review article:

A PARADIGM SHIFT IN THE DETECTION OF BLOODBORNE PATHOGENS: CONVENTIONAL APPROACHES TO RECENT DETECTION TECHNIQUES

Sonali Khanal[1], Manjusha Pillai[1], Deblina Biswas[2], Muhammad Torequl Islam[3,4,5], Rachna Verma[6,7,*], Kamil Kuca[7,8,9], Dinesh Kumar[1], Asim Najmi[10], Khalid Zoghebi[10], Asaad Khalid[11,*], Syam Mohan[12,13]

1 School of Bioengineering and Food Technology, Shoolini University of Biotechnology and Management Sciences, Solan 173229, India
2 Instrumentation and Control Engineering, Dr. B. R. Ambedkar National Institute of Technology Jalandhar, Punjab, 144011, India
3 Department of Pharmacy, Bangabandhu Sheikh Mujibur Rahman Science and Technology University, Gopalganj 8100, Bangladesh
4 Department of Pharmacy, Bangabandhu Sheikh Mujibur Rahman Science and Technology University, Gopalgonj 8100, Bangladesh
5 Bioinformatics and Drug Innovation Laboratory, BioLuster Research Center Ltd., Gopalganj 8100, Bangladesh
6 School of Biological and Environmental Sciences, Shoolini University of Biotechnology and Management Sciences, Solan 173229, India
7 Faculty of Science, University of Hradec Kralove, Rokitanskeho 62, 500 03 Hradec Kralove, Czech Republic
8 Biomedical Research Center, University Hospital Hradec Kralove, 50003 Hradec Kralove, Czech Republic
9 Center for Advanced Innovation Technologies, VSB-Technical University of Ostrava,70800, Ostrava-Poruba, Czech Republic
10 Department of Pharmaceutical Chemistry and Pharmacognosy, College of Pharmacy, Jazan University, Jazan 45142, Saudi Arabia
11 Health Research Center, Jazan University, P. O. Box 114, Jazan, 82511, Saudi Arabia
12 Center for Global Health Research, Saveetha Medical College and Hospitals, Saveetha Institute of Medical and Technical Sciences, Saveetha University, India
13 School of Health Sciences, University of Petroleum and Energy Studies, Dehradun, Uttarakhand, India

* **Corresponding authors:** Rachna Verma, School of Biological and Environmental Sciences, Shoolini University of Biotechnology and Management Sciences, Solan 173229, India. Tel.: +91 98162 91543, E-mail: rachnac83@gmail.com
Asaad Khalid, Health Research Center, Jazan University, P. O. Box 114, Jazan, 82511, Saudi Arabia. E-mail: drasaad@gmail.com

https://dx.doi.org/10.17179/excli2024-7392

This is an Open Access article distributed under the terms of the Creative Commons Attribution License (http://creativecommons.org/licenses/by/4.0/).

Supplementary Table 1: Common human bloodborne pathogens and the diseases they cause

Class	Bloodborne pathogens	Diseases caused
Viruses	• Human Cytomegalovirus- HHV5	• Infectious mononucleosis
	• Human Immunodeficiency Virus	• Acquired Immunodeficiency syndrome
	• Hepatitis B	• Chronic liver infection
	• Hepatitis C	• Chronic liver infection
	• Hepatitis A	• Contagious liver infection
	• West Nile Virus	• Encephalitis, meningitis, or meningoencephalitis
	• Dengue Virus	• Dengue fever
	• Epstein-Barr virus (EBV)	• Mononucleosis
Fungus	• *Candida albicans*	• Candidiasis
	• *Candida parapsilosis*	• Sepsis and tissue infections
	• *Candida glabrata*	• Urogenital tract infections
	• *Cryptococcus*	• Cryptococcosis
	• *Histoplasma*	• Histoplasmosis
	• *Aspergillus species*	• Aspergillosis
Bacteria	• *Staphylococcus aureus (Gram +)*	• Sepsis and endocarditis
	• *Staphylococcus epidermidis (Gram +)*	• Prosthetic joint infections and valve endocarditis
	• *Escherichia coli (Gram -)*	• Sepsis, neonatal meningitis, urinal tract infections
	• **Enterococcus faecalis**	• Bacteremia and Endocarditis
	• **Enterococcus faecium**	• Intra-abdominal infection and endocarditis
	• **Streptococcus pneumoniae**	• Pneumococcal disease
	• **Haemophilus influenzae**	• Meningitis and Bacteremia
	• **Acinetobacter baumannii**	• Pneumonia and bacteremia
	• **Brucella abortus**	• Brucellosis
Parasites	• *Babesia microti & Babesia divergens*	• Babesiosis
	• *Trypanosoma cruzi*	• Chagas disease
	• *Leishmania donovani*	• Leishmaniasis
	• *Plasmodium berghei*	• Malaria
	• *Treponema pallidium*	• Syphilis

EXCLI Journal 2024;23:1245-1275 – ISSN 1611-2156
Received: May 15, 2024, accepted: September 04, 2024, published: October 17, 2024

Supplementary Table 2: Classification of bloodborne pathogens prevalent in food animals

Food Animals	Blood parasites	Virus	Bacteria	Fungus
Goat	*Theileria hirci*<br>*Theileria ovis*<br>Theileria separata<br>*Eperythrozoon ovis*<br>**Babesia ovis**<br>**Anaplasma ovis**	Rift Viral Fever	*Ehrlichia ruminantium*<br>**Anaplasma marginale**<br>COXIELLA BURNETII	*Aspergillus sp.*<br>*Candida sp.*
Sheep	*Theileria hirci*<br>*Theileria ovis*<br>*Theileria separata*<br>*Eperythrozoon ovis*<br>*Babesia motasi*<br>*Anaplasma ovis*	Rift Viral Fever	*Anaplasma phagocytophilum*	*Aspergillus sp.*<br>*Candida sp.*
Cattle	*Theileria annulate*<br>*Theileria sp*<br>*Eperythrozoon wenyoni*<br>*Babesia bigemina*	Bovine leukosis virus	*Haemotrophic mycoplasma*<br>*Anaplasma marginale*<br>*Anaplasma centrale*	**Trichophyton verrucosum**
Chicken	*Leucocytozoon caulleryi*<br>*Leucocytozoon sabrazesi*,<br>*Leucocytozoon schoutedeni*<br>*Plasmodium gallinaceum*,<br>*Plasmodium juxtanucleare*<br>*Plasmodium durae*<br>*Isospora*	Avian infuenza	*Aegyptianella pullorum*	*Cryptococcus neoformans*<br>*Histoplasma capsulatum*
Pig	**Babesia trautmanni**<br>**Babesia perroncitoi**<br>**Eperythrozoon suis**<br>**Eperythrozoon parvum.**<br>**Trypanosoma** spp<br>**Anaplasma** spp	African swine fever virus	*Brucella suis*	*Aspergillus sp.*
Fish	HAEMOGREGARINA BIGEMINA<br>*Cryptobia*<br>*Trypanosoma*	Cyprinid herpesvirus-3 (CyHV-3),	*Aeromonas spp.,*<br>*Mycobacterium,*<br>*Salmonella spp., Streptococcus spp.,*<br>*Vibrio spp*	*Branchiomyses sanguinis*<br>*Ichthyophonus hoferi*
